# Extreme lithium isotopic fractionation in three zircon standards (Plešovice, Qinghu and Temora)

**DOI:** 10.1038/srep16878

**Published:** 2015-11-23

**Authors:** Yu-Ya Gao, Xian-Hua Li, William L. Griffin, Yan-Jie Tang, Norman J. Pearson, Yu Liu, Mei-Fei Chu, Qiu-Li Li, Guo-Qiang Tang, Suzanne Y. O’Reilly

**Affiliations:** 1 State Key Laboratory of Lithospheric Evolution, Institute of Geology and Geophysics, Chinese Academy of Sciences, Beijing 100029, China; 2 ARC Centre of Excellence for Core to Crust Fluid Systems and GEMOC, Department of Earth and Planetary Sciences, Macquarie University, NSW 2019, Australia; 3 College of Earth Science, University of Chinese Academy of Sciences, Beijing 100049, China; 4 Department of Geosciences, National Taiwan University, Taipei 10617, Taiwan

## Abstract

To understand the behavior of Li in zircon, we have analyzed the abundance and isotopic composition of Li in three zircon standards (Plešovice, Qinghu and Temora) widely used for microbeam analysis of U-Pb ages and O-Hf isotopes. We have mapped Li concentration ([Li]) on large grains, using a Cameca 1280HR Secondary Ion Mass Spectrometer (SIMS). All zircons have a rim 5–20 μm wide in which [Li] is 5 to 20 times higher than in the core. Up to ~20‰ isotopic fractionation is observed on a small scale in the rims of a single zircon grain. The measured δ^7^Li values range from –14.3 to 3.7‰ for Plešovice, –22.8 to 1.4‰ for Qinghu and –4.7 to 16.1‰ for Temora zircon. The [Li] and δ^7^Li are highly variable at the rims, but relatively homogenous in the cores of the grains. From zircon rim to core, [Li] decreases rapidly, while δ^7^Li increases, suggesting that the large isotopic variation of Li in zircons could be caused by diffusion. Our data demonstrate that homogeneous δ^7^Li in the cores of zircon can retain the original isotopic signatures of the magmas, while the bulk analysis of Li isotopes in mineral separates and in bulk-rock samples may produce misleading data.

Lithium (Li) isotopes are a potentially powerful tracer in a wide range of geochemical processes because of their large relative mass difference (ca 16.7%), and the broad elemental dispersion and moderate incompatibility^1^,^2^,^3^,^4^,^5^,^6^. Li is highly soluble and very sensitive to fluid/melt–mineral interactions, and can display up to 80‰ isotopic fractionation in natural systems^3^,^7^. However, the factors that make Li geochemically useful also make the quantification and interpretation of its isotopic composition extraordinarily challenging^3^. It is still controversial to what extent Li isotopic signatures and abundances can be preserved in whole-rock samples and minerals, as there are major differences between whole-rock and *in situ* results. While the overall spread in δ^7^Li values is similar in granitoids world-wide, with whole-rock δ^7^Li ranging from –5 to +10‰^6^, zircons from Jack Hills metasedimentary rocks show a wider range of δ^7^Li values, over 30‰^2^. This contrast in Li isotopic variations between granitoid whole-rocks and zircons (which predominantly have crystallized from granitoid magmas) has aroused great interest. Cherniak and Watson (2010)^8^ conducted experiments on Li diffusion in zircon at different temperatures and with different fluid compositions. Their results indicated that Li diffusion is slower in zircon than in other major rock-forming minerals, but it still diffuses at a considerable rate. However, direct comparisons between Li isotope compositions in whole-rock samples and zircon have yet been undertaken to understand their relationship.

In this study, we have analyzed Li isotopes (δ^7^Li) and abundance ([Li]), in both whole-rock samples and *in situ* in zircon, and have imaged the distribution of seven trace elements (Li, P, Ti, Yb, Y, U, and Th), in three well-characterized zircon standards commonly used for U-Pb and Hf-O isotopic analysis. Our aim is to shed light on the Li-isotope characteristics of these zircons, to find out whether variations in the Li isotopes reflect differences in source values and/or is caused by later diffusion. This is fundamental to evaluating the feasibility of using Li-isotope analysis of whole-rock samples and zircon as a geochemical tracer to identify the source regions of magmas.

## Sample Information

Three standard zircons (Plešovice^9^, Qinghu^10^ and Temora^9^,^11^,^12^) and have been chosen for this study, as they (i) are homogeneous and concordant in terms of radiogenic Pb/U ratios, (ii) are homogeneous in Hf and O isotopic composition, (iii) have crystalline (nonmetamict) structures, and (iv) occur as mm-sized grains. The M257 zircon^13^,^14^,^15^,^16^ is used as zircon Li-isotope analysis standard. The isotopic characteristics of the zircons are summarized in Table 1.

### Plešovice

Plešovice zircons were extracted from a high-temperature potassic granulite in the southern Bohemian Massif of the Czech Republic. The content of zircon in the granulite is up to 0.6 wt%, and it is variable on the scale of tens of centimeters. The Plešovice zircon is homogeneous in terms of U-Pb age and Hf isotopes, and thus is widely used as a standard for microbeam isotopic analyses^9^. Zircon grains are prismatic, pale pink to brown euhedral crystals and have been tested for Li-isotope homogeneity in a previous study, which showed highly heterogeneous Li-isotope compositions (~10‰ variation in δ^7^Li)^16^. Two grains (maximum lengths of 2094 μm and 1770 μm, respectively) were selected and mounted to acquire detailed diffusion profiles.

### Qinghu

The Qinghu monzonite is located in the southwestern part of the Nanling Range in south China^17^. The rock types of the pluton are coarse-grained hornblende monzonite, medium-grained quartz-bearing hornblende monzonite and fine-grained quartz monzonite^10^. Zircons from the Qinghu monzonite are euhedral, range mostly from 100 to 300 μm in length, and have length/ width ratios mostly between 1:2 and 1:4. Most zircons show a clear euhedral concentric zoning in cathodoluminscence (CL). Previous studies indicate that Qinghu zircon is quite homogeneous and can be used as a working reference standard for SIMS U-Pb and Hf-O isotopic analysis^10^.

### Temora

The Temora zircons were extracted from the Middledale Gabbroic Diorite in the Tasman Oragenic Belt, Australia. The rock is medium- to coarse-grained, relatively unaltered and composed dominantly of labradoritic plagioclase and brown pargasitic hornblende (partially replaced by chlorite)^18^. Zircons are mostly euhedral, and 100–550 microns in length. Temora zircons have been widely used as standards for U-Pb age and Hf-O isotope microanalyses^9^,^11^,^12^,^19^.

## Results

Zircons with euhedral crystal shapes were analyzed for both Li isotopic composition and element distribution. The abundance and isotopic composition of Li were measured along 12 traverses across seven zircon grains (2 Plešovice, 4 Qinghu and 1 Temora). Nine ion-imaging analyses were used to partially map the zircon grains. Traverses of 5–15 analytical spots at 10–200 μm intervals were performed across selected grains in order to investigate the degree of homogeneity. A smaller primary ion beam (∼5 μm) was used to conduct a more thorough study of the rim phenomena. Detailed information for all the measurements is listed in the Appendix dataset. Generally, all of the zircon grains have a 5–20 μm wide rim with extremely high [Li], about 5 to 20 times higher than the core. There also is extremely large isotopic fractionation (~20‰) at the zircon rims on a small scale (~50 μm).

Negative correlations between [Li] and δ^7^Li were encountered at the rim of the grains. Variability in δ^7^Li, related to cracks with higher Li contents, was also observed and these spots were eliminated.

### Plešovice

**Figure 1:**
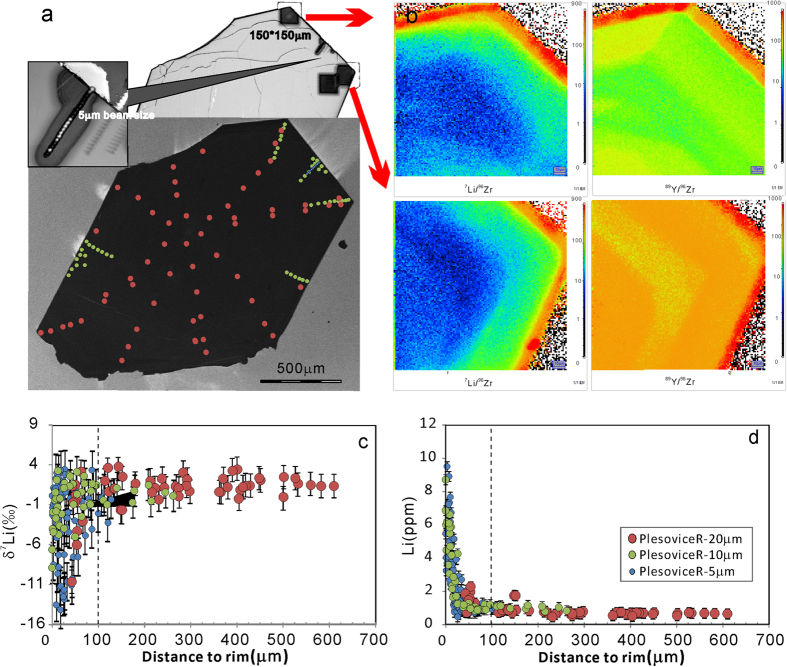
(**a**) Trace outlines on the CL image of Plešovice-R to show the position of the Li isotopic and [Li] measurements by SIMS. (**b**) The ion image of the variation of Li and Y content (bright colors = higher concentration). (**c**) δ^7^Li *vs* relative distance to the rim. (**d**) [Li] *vs* relative distance to the rim. Uncertainty of standard analysis (2SD) has been shown in the plots while 2SE of individual analyses is smaller than 2SD. Typical analytical uncertainty of δ^7^Li is ∼2‰ for the 20 μm pits, ~2.5‰ for the 10 μm pits and ∼5‰ for 5 μm pits (2 SD). Distance to rim is calculated by the X-Y position of the analytical spot to the nearest rim.

**Figure 2:**
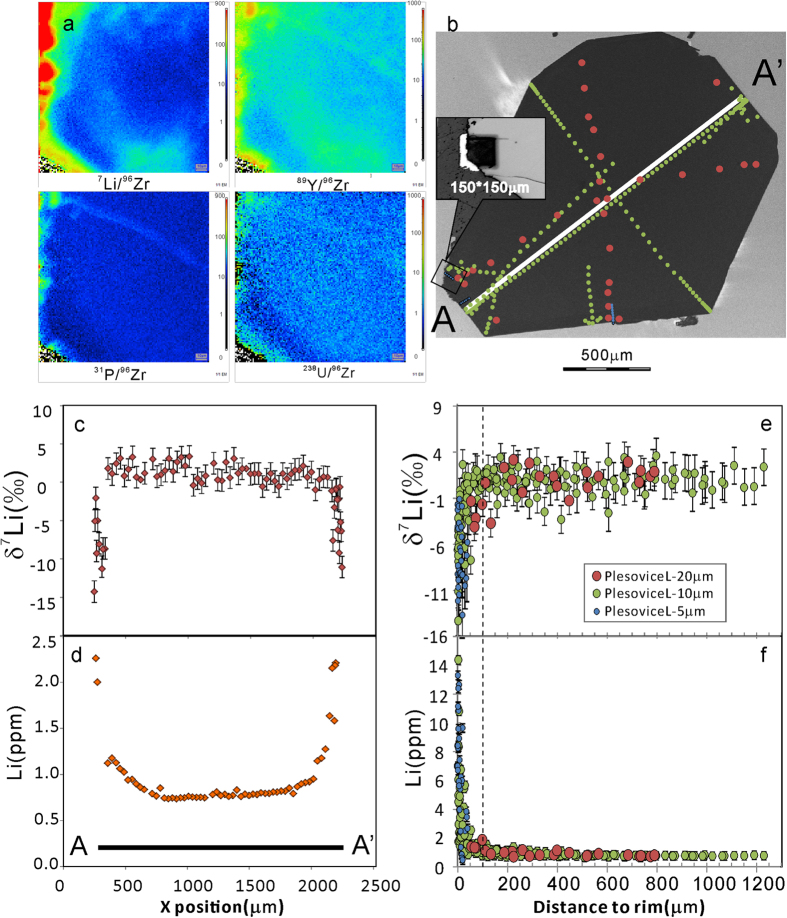
(**a**) Ion image of the variation of Li, Y, P and U content of Plešovice-L. (**b**) Trace outlines on the CL image to show the position of the Li isotopic and [Li] measurements. (**c**) δ^7^Li *vs* X position of AA’ profile. (**d**) [Li] *vs* X position of AA’ profile. (**e**) δ^7^Li *vs* relative distance to the rim. (**f**) [Li] *vs* relative distance to the rim.

Four whole-rock samples of Plešovice have been analyzed (Table 2); their [Li] is ~54 ppm, and δ^7^Li ranges from −1.6 to −0.6‰ (±0.5, 2SD). Two Plešovice megacrystic zircons (Plešovice-L & R) have been studied in detail. ~20 μm, ~10 μm and ~5 μm beam spots were used to traverse each grain. [Li] profiles across the two megacrysts are nearly flat at the center, but rise dramatically at the rim (Figs 1 and 2). The overall [Li] ranges from 0.5 to 14.4 ppm. The δ^7^Li values range from −14.3 to +3.7‰. Fractionation of Li isotopes is up to 18‰ in a single grain. Both Plešovice megacrysts show a plateau of constant δ^7^Li in their cores, but striking heterogeneity at the rims.

For the Plešovice-R zircon (Fig. 1), [Li] ranges from 0.5 to 9.5 ppm and δ^7^Li ranges from −14.1 to +3.7‰. The δ^7^Li values in the rim of Plešovice-R show very complex spatial variation; therefore, ~10 m and ~5 μm beam spots were used to map the changes in Li isotopes over small areas. The ion image is a visual display of changes in [Li] and [Y] at the rim. Both [Li] and δ^7^Li are uniform at distances of >100 μm from the rim (Fig. 1). The Plešovice-L zircon (Fig. 2) has [Li] ranging from 0.5 to 14.4 ppm and δ^7^Li from −14.3 to +3.7‰. The ion image displays [Li] and [Y] at a broken rim. Profile AA’ along the zircon demonstrates that the Li isotopes change across the entire zircon. [Li] shows a rim-ward increase, while δ^7^Li is negatively correlated with [Li] and characterized by a typical diffusion profile. Both [Li] and δ^7^Li become uniform at distances of >100 μm from the rim (Fig. 2). Compared to Plešovice-R, Plešovice-L has a more euhedral shape and fewer cracks, and δ^7^Li variations at the rim of Plešovice-L are less complex than in Plešovice-R.

### Qinghu

The whole–rock [Li] of the Qinghu monzonite is ~23 ppm; δ^7^Li values range from 0.9 to +2.5‰. Zircon grains have been traversed using both ~20 μm and ~10 μm beams. [Li] ranges from 0.3 to 11.8 ppm and δ^7^Li values range from −22.8 to +1.4‰ (Table 3). Compared to the Plešovice samples, Qinghu zircons have higher [Li] in the cores, mostly around 1–2 ppm, and extremely high [Li] in their rims, 6–15 times higher than in the core.

**Figure 3:**
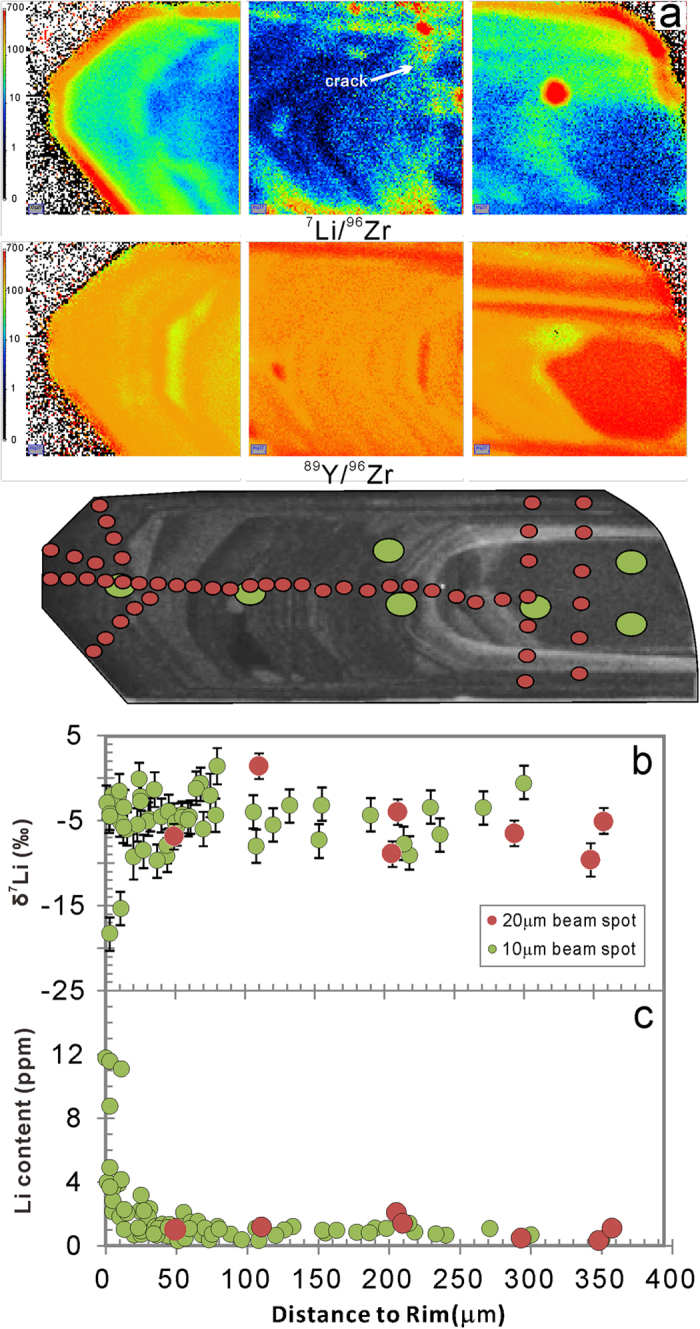
(**a**) Ion image of the variation of Li, Y content and CL image of the Qinghu zircon. (**b**) δ^7^Li *vs* relative distance to the rim. (**c**) [Li] *vs* relative distance to the rim.

Across the Qinghu_1 zircon, [Li] ranges from 0.3 to 11.8 ppm and δ^7^Li from −18.3 to +1.4‰. The ion image indicates that [Li] and [Y] are to some extent correlated. It also shows higher [Li] near cracks while [Y] remains unaffected. The [Li] of Qinghu_1 increases rimward. The δ^7^Li values in the rim, obtained using the ~20 μm and ~10 μm beam spots, are negatively correlated with [Li] and define a typical diffusion profile (Fig. 3).

**Figure 4:**
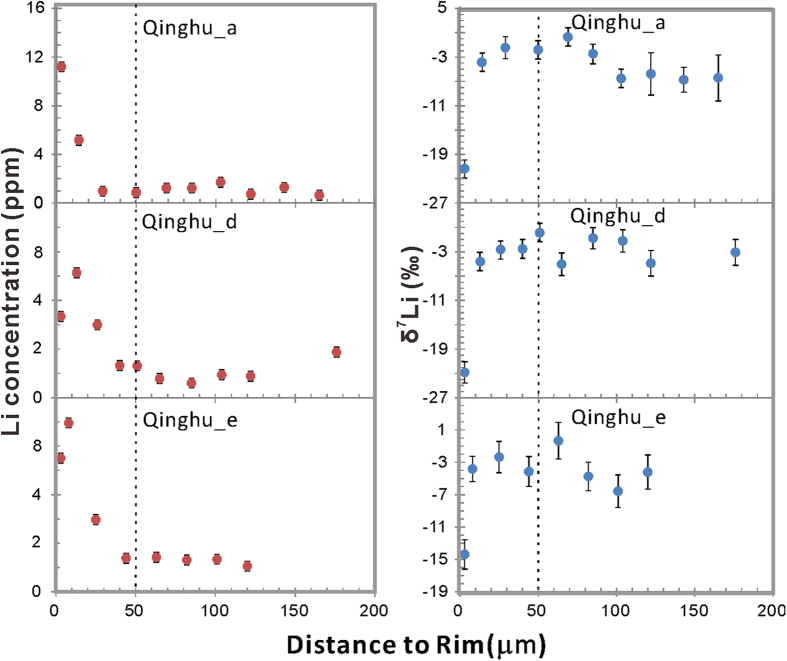
Plots of δ^7^Li and [Li] *vs* relative distance to the rim of the Qinghu zircons.

Another three Qinghu zircons were traversed using a ~10 μm beam spot. Overall, their [Li] shows a rimward increase and becomes relatively uniform at distances from rim of >60 μm (Fig. 4). Generally, the δ^7^Li values are higher and more homogeneous in the core than at the rim.

### Temora

**Figure 5:**
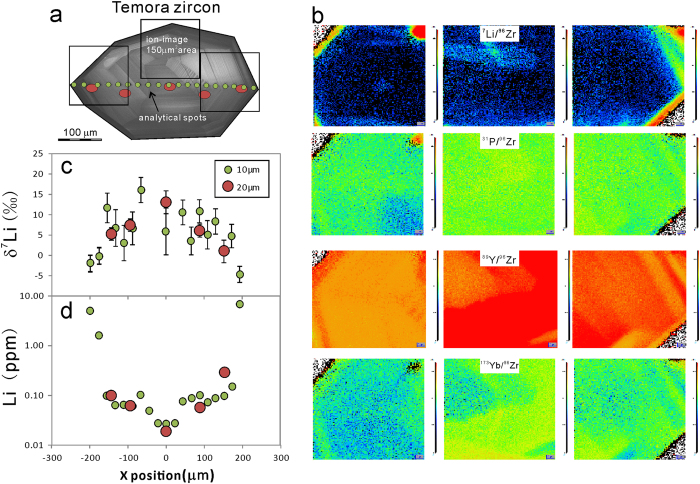
(**a**) The CL image and analytical spots of the Temora zircon. (**b**) ion image of Li, P, Y, and Yb. (**c**) δ^7^Li profile through zircon. (**d**) [Li] profile through zircon.

The whole–rock [Li] of Temora is ~11.4 ppm, and δ^7^Li values range from 0.7 to 1.5‰. The Temora zircon grains have been traversed using 20 μm and 10 μm beam sizes. All the data are shown in the Appendix dataset. Ion images show the distributions of Li, P, Y, Yb (Fig. 5). Y and Yb are positively correlated with [Li] while P is quite homogenous from the rim to core.

Temora zircons have quite low Li contents, which leads to larger analytical errors. For all twenty-four analytical spots, [Li] ranges from 0.02–6.84 ppm and δ^7^Li from −4.7 to +16.1‰. There is a typical diffusion profile with gradually decreasing [Li] and increasing δ^7^Li from rim to core (Fig. 5c,d). Except for the spots with very low [Li], the δ^7^Li is relatively homogeneous (with a weighted average of 7.0 ± 1.8‰) in the centers of grains at least 150 μm across.

## Discussion

To date, only a few studies on the isotopic composition of Li in zircons have been reported^2^,^8^,^16^,^20^. The results of these studies are by no means conclusive with respect to either diffusion fractionation, or isotopic fractionation between zircon and melt or zircon and surrounding minerals. Significant Li-isotope heterogeneity occurs on both intra- and inter-granular levels. There is up to ~20‰ isotopic fractionation within ~50 microns of the rim of a single grain (Figs 1, 2, 3, 4). Extreme Li-isotope fractionation (~35‰, ~48‰) also has been observed in olivine^21^,^22^ and orthopyroxene (~40‰)^23^. Such extreme fractionation is thought to be produced by disequilibrium fractionation. The processes responsible may include metasomatism of mantle rocks by silicate and carbonatite melts and hydrous fluids^21^, preservation or modification of fluid/melt interactions in recycled oceanic crust^22^, or diffusion-driven fractionation and emplacement-related alteration coupled with isotopic exchange^23^.

Our data display the largest intra-granular fractionations (~20‰) found in published zircon Li-isotope data. To confirm the robustness of the data at the rims, we examined the Li isotopes in the epoxy and in the rim of the sloped surface of the M257 standard in order to check the following possibilities: 1) contamination by the epoxy; 2) analytical artifacts from sample geometry and topography^24^,^25^,^26^,^27^. All the analysis spots are within the zircon grains, so that the epoxy has no effect on the analysis of the zircon rims. Moreover, none of the [Li] and isotopic data of zircons, olivines and pyroxenes analyzed previously in our lab show any effect of epoxy on the rim analyses of mineral grains^16^,^21^,^22^,^28^,^29^,^30^.

The charge effect was carefully avoided during the analysis. The sample mount has been taken out and re-coated if the analytical spots were too close to each other. Moreover, data have been screened by ion yield and analytical errors. The high-[Li] zones at the rim are not observed in the M257 standard zircon, which is a fragment, and are not observed where the zircon grain is bounded by a broken surface. It is only seen at growth surfaces (crystal faces). Since both types of grain boundary stand up from the epoxy mount, the high-[Li] zones cannot be ascribed to a topographic effect; therefore, they represent processes affecting the original boundary between the zircon crystal and its host rock. Li is also concentrated in fissures and interstices of the zircon grains. Considering the high Li abundance in the corresponding whole-rocks, the high [Li] and extreme δ^7^Li variations at the zircon rims are most likely caused by a gradient in [Li] via diffusion of Li from Li-rich mineral phases on cracks and surfaces, and/or a fluid phase with high δ^7^Li. The diffusive fractionation of Li isotopes can occur during cooling or in relation to overgrowth, which is controlled by variable mineral-melt and mineral-mineral partitioning of Li. The zircon standards have good crystalline structure and are homogeneous in terms of radiogenic Pb/U ratios and Hf-O isotopic composition. It is reasonable to conjecture that whole zircon grains were most likely homogeneous in Li isotopes during their crystallization, and in equilibrium with the host magma. The Li isotopes of zircon rims thus were affected by late diffusion-driven processes. This process might have been a “two-stage diffusion”: 1) Diffusion between melt and zircon: zircon crystallized in equilibrium with magma, then Li diffused from the melt into zircon grains during magmatic cooling, driven by the [Li] gradient and the increasing partition coefficient of Li between zircon and melt; 2) Diffusion between solid mineral phases: after the melt fully crystallized, Li could diffuse from the adjacent minerals with high [Li] (feldspar/biotite/quartz, etc.) into zircon grains.

**Figure 6:**
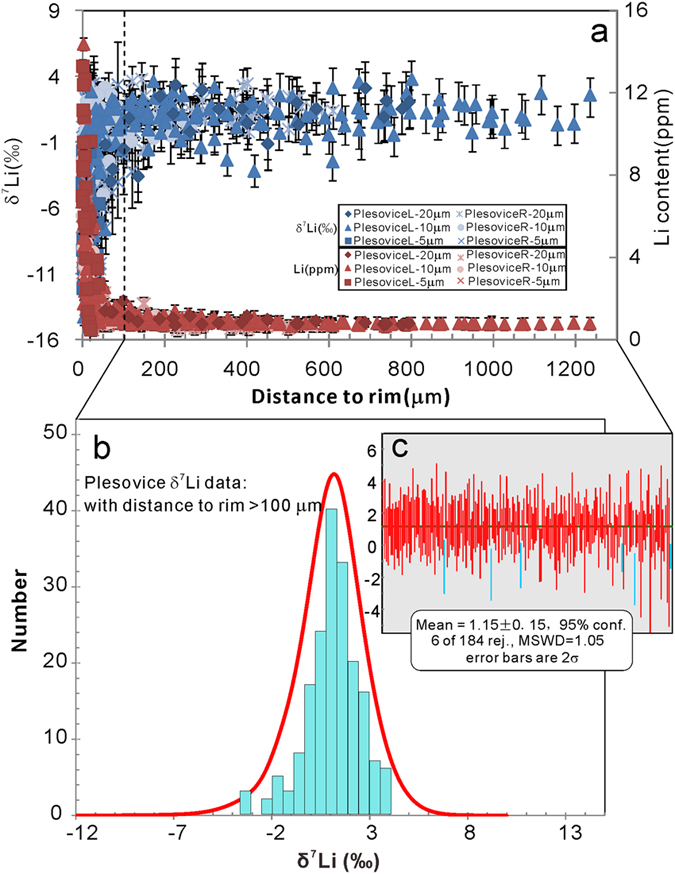
(**a**) Compiled data for all the analysed spots on the Plešovice zircons. (**b**) Probability density plot and (**c**) Weighted average of analysed spots more than 100 μm from the rim.

The preservation of the original Li isotopic data in zircon will depend on the cooling rate and the size of zircon grains^8^. Slow cooling rates might greatly modify the original “equilibrium” Li isotopes. In spite of this, if the zircon grain is large enough or the diffusion period is short enough, the Li isotopes in the core will retain the pristine δ^7^Li signature of the melt from which the zircon crystalized. For large zircon grains like Plešovice-R and -L, the δ^7^Li in the central parts of the grains shows a plateau of constant δ^7^Li which can represent the original δ^7^Li values of the zircon. Figure 6 presents the compiled data of all the analyzed spots on the Plešovice zircons. The analyzed spots more than 100 μm from the rim show a good Gaussian distribution, with weighted average δ^7^Li = 1.15 ± 0.15‰ (1σ). This should be the best estimate of the pristine Li-isotope composition of the Plešovice zircon. The Plešovice granulite was derived from the mature crustal sources by Sr-Nd^31^ and Hf-O isotopes^15^ and is consistent with zircon δ^7^Li.

It is noteworthy that Li isotopes from large zircon cores retain the initial Li signature, but they are more or less different from the whole-rock δ^7^Li. The cores of Plešovice zircon have higher δ^7^Li values (0.1‰ to 1.4‰) than the whole-rock (−1.6‰ to − 0.6‰) while the δ^7^Li of most Qinghu zircon cores (−6.4‰ to −3.6‰) is lower than the whole-rock values (0.9‰ to 2.5‰). Studies of Li-isotope fractionation in peridotites and basalts show little fractionation of Li isotopes at high temperatures during partial melting and equilibrium fractionation^20^,^23^,^32^. Bouvior (2007)^20^ assumed that Li-isotope fractionation between melt and zircon is less than 2‰. Therefore, the original δ^7^Li value of whole-rock is likely to be similar to the original value in zircons. However, as Li is a highly mobile element and has high diffusivity, the initial Li-isotope signature of the whole-rock is unlikely to survive through later geological processes such as fluid/melt interaction, high-grade metamorphism and hydrothermal alteration. Therefore, we propose that the bulk-rock data are misleading because they are a weighted average of Li in all rock-forming and accessory minerals, and could reflect the integrated effects of multiple geological processes. The inconsistent δ^7^Li results between whole-rocks and zircon cores are most likely indicative of later processes, for example fluid addition after zircon crystallization, which could shift the Li isotopic composition of whole rock.

As the Plešovice, Qinghu and Temora zircons are homogeneous in terms of U-Pb ages and O-Hf isotopes, one can assume their parental melts remained homogeneous and no separate fluid/melt was added during the crystallization of the zircons. Therefore, the plateau of [Li] and δ^7^Li in the core of large Plešovice zircons could reflect the pristine Li-isotope composition of the magma from which zircon crystallized. Unlike the equant Plešovice zircons, the Qinghu zircons are elongated and have length to width ratios of ~3:1 to 4:1. The data were collected along long axis of the zircon while the plateau is obtained more than 60 μm from the rim, which is larger than the half-width of the short axis. Therefore, the plateau δ^7^Li data of the Qinghu zircon is more likely to be compromised by Li diffusion parallel to the short axis and cannot represent the primary zircon δ^7^Li. Moreover, the Hf-O isotopes of the Qinghu zircon suggest it originated from a depleted-mantle source with little crustal contamination and it therefore should have a positive δ^7^Li, close to the normal mantle, which is inconsistent with the negative δ^7^Li found in this study. This implies that the δ^7^Li of Qinghu zircons have been lowered by later processes. Therefore, if zircon grains are small (e.g. Temora zircon), the δ^7^Li value in the center of the grain may have been shifted by diffusion and/or later processes and thus cannot represent the pristine Li-isotope value. Confirmation of homogeneous δ^7^Li and [Li] by multiple analyses is necessary to define the pristine igneous Li signature in zircon. Heterogeneity (δ^7^Li variations ~20‰ within individual grains) indicates that the bulk analysis of Li isotopes in mineral separates and in bulk-rock samples may produce misleading data.

## Methods

The Li-isotope composition of whole-rock samples was measured using solution MC-ICP-MS methods at the Geochemical Analysis Unit, GEMOC, Macquarie University. The procedure used for purification of the dissolved solution is similar to those previously described by Seitz *et al.* (2004)^33^. The isotopic analysis of each unknown sample was bracketed by two 15 ppb L-SVEC standard solutions (^7^Li/^6^Li = 12.039 ± 0.03)^34^ to correct for mass fractionation. The raw δ^7^Li values of unknowns are expressed as δ^7^Li = [(R_measure_/R_L-SVEC_)–1]*1000‰. The accuracy of the results was monitored using international rock standard RGM-1 with average δ^7^Li values of 2.3 ± 0.4‰(2SD) in agreement with the published values^35^. The average reproducibility based on samples and standards is ±0.9‰ (2SD). The analytical data are listed in Table 2.

SIMS *in situ* analysis of Li isotopes were performed using the Cameca IMS 1280HR SIMS at the Institute of Geology and Geophysics, Chinese Academy of Sciences (IGG–CAS) in Beijing, following the procedures described by Li *et al* (2011)^16^. A ~20 μm and ~10 μm beam was used to traverse zircon grains, while a ~5 μm beam was used for detailed analysis at the zircon rim. Drift of δ^7^Li values in the M257 standard over repeated analyses is typically <2‰ in a single analytical session with an internal precision between 1.5 and 2.0‰ (2SD) for a single spot with ~20 μm beam size, 2.5‰ for ~10 μm beam size and 5.0‰ for ~5 μm beam size. Measurements of Li isotopes and concentrations were conducted in several separate sessions. The full data set is presented in Appendix dataset.

Secondary ion images were obtained for construction of element concentration maps (Li, P, Ti, Yb, Y, U, and Th) over selected zircon grains within a single analysis using a peak-switching mode. The sample was pre-sputtered over a 200 μm × 200 μm area by rastering for 300 s in order to eliminate surface contamination. We used a focused beam of O^−^ with a diameter of 5 μm (∼1 nA) to scan a 150 μm × 150 μm area. The Dynamic Transfer Optical System was applied to obtain a wide field of view and ^7^Li^+^ was detected by the electron multiplier. Other mass spectrometric conditions were similar to those used for isotopic analyses of the samples.

## Additional Information

**How to cite this article**: Gao, Y.-Y. *et al.* Extreme lithium isotopic fractionation in three zircon standards (Plešovice, Qinghu and Temora). *Sci. Rep.* **5**, 16878; doi: 10.1038/srep16878 (2015).

## Supplementary Material

Supplementary Appendix Dataset

## Figures and Tables

**Table 1:** Standards information^9^,^10^,^11^,^12^,^13^,^14^,^15^,^16^,^17^,^18^,^19^.

	M257	Plesovice	Qinghu	Temora
Rock type	gem	granulite	syenite	diorite
Zircon Radius	megacryst	megacryst	~400 μm	~400 um
Age (Ma)	561.3 ± 0.3	337.1 ± 0.4	159.5 ± 0.2	416.8
^176^Hf/^177^Hf	0.281544 ± 18	0.282482 ± 13	0.283002 ± 04	0.282686 ± 08
δ^18^O (‰)	13.9 ± 0.1	8.2 ± 0.2	5.4 ± 0.2	8.1

**Table 2:** Whole rock Li-isotope results.

	Li (ppm)	δ^7^Li (‰)	±σ
Plesovice-1	56.6	−0.6	0.3
Plesovice-2	54.6	−1.6	0.2
Plesovice-3	50.6	−1.5	0.3
Plesovice-4	52.7	−1.4	0.1
Qinghu-1	21.4	2.0	0.6
Qinghu-2	21.0	0.9	0.4
Qinghu-3	25.6	2.5	0.1
Qinghu-4	27.1	2.4	0.3
Qinghu-5	20.0	1.8	0.1
**Temora-1**	11.9	0.7	0.3
**Temora-2**	10.8	1.5	0.1

**Table 3:** Summary of SIMS Li isotopes for three zircon standards.

		δ^7^Li (‰)					Li (ppm)			
Sample name	Spot size	Min	Max	Fractionation	core average*	1sd	Min	Max	core average*	1sd
PlesoviceR	(20 μm)	−10.7	3.7	14.8	1.4	1.2	0.5	2.3	0.7	0.2
	(10 μm)	−8.9	3.3	12.2	0.1	0.9	0.8	8.7	1.0	0.1
	(5 μm)	−14.1	3.4	17.6	–	–	0.5	9.5	–	–
PlesoviceL	(20 μm)	−4.0	3.3	7.3	1.0	1.7	0.7	1.9	0.9	0.3
	(10 μm)	−14.3	3.7	18.0	1.0	1.2	0.7	14.4	0.9	0.2
	(5 μm)	−13.7	−1.0	12.7	–	–	0.5	13.3	–	–
Plesovice-inter grains		−14.3	3.7	18.0	–	–	0.5	14.4	–	–
Qinghu-1	(20 μm)	−9.6	1.4	11.0	−5.5	4.0	0.3	2.1	1.1	0.6
	(10 μm)	−18.3	−0.1	18.2	−5.7	3.9	0.3	11.8	1.9	2.5
	(5 μm)	−9.3	1.4	10.7	–	–	0.7	4.0	–	–
Qinghu-a	(10 μm)	−21.4	0.3	21.6	−6.4	0.4	0.6	11.2	1.1	0.5
Qinghu-d	(10 μm)	−22.8	0.1	22.9	−3.6	1.3	0.6	5.1	1.2	0.4
Qinghu-e	(10 μm)	−14.4	−0.3	14.1	−5.4	1.7	1.1	7.0	1.2	0.2
Qinghu-inter grains		−22.8	1.4	24.2	–	–	0.3	11.8	–	–
Temora	(20 μm)	1.1	13.1	12.0	–	–	0.0	0.3	–	–
	(10 μm)	−4.7	16.1	20.8	–	–	0.0	6.8	–	–

*Data with distance to rim >100 μm.
